# Case Report: ^18^F-Fluorocholine PET/CT for detecting brown tumours in patients with hyperparathyroidism: a short case series and literature review

**DOI:** 10.3389/fnume.2025.1693642

**Published:** 2025-11-07

**Authors:** J. Zhang-Yin, Emmanouil Panagiotidis

**Affiliations:** 1 Department of Nuclear Medicine, Clinique Sud Luxembourg, Arlon, Belgium; 2 Department of Nuclear Medicine, Tenon Hospital, APHP, Paris, France; 3 Department of Nuclear Medicine, University Hospital of Larissa, Faculty of Medicine, University of Thessaly, Larissa, Greece

**Keywords:** ^18^F-Fluorocholine PET/CT, brown tumours, hyperparathyroidism, osteolytic bone destruction, parathyroid adenoma

## Abstract

**Introduction:**

Brown tumours (BTs), also known as osteitis fibrosa cystica, are benign osteolytic lesions associated with hyperparathyroidism (HPT). BTs are cured by correcting the hypercalcaemia and HPT, which often requires surgical resection of the parathyroid adenoma. ^1^⁸F-fluorocholine (FCH) PET/CT is becoming an effective tool for detecting parathyroid adenomas in cases of hyperparathyroidism. This study aims to evaluate the role of FCH PET/CT in detecting brown tumours in patients with hyperparathyroidism.

**Clinical cases:**

Three cases are presented to demonstrate the effectiveness of FCH PET/CT in detecting BTs in patients presenting with clinical and biochemical signs of HPT with suspicion of BTs.

**Literature review:**

A literature review aimed to summarize the bibliographic evidence on the use of this technique in this setting, which is relatively innovative.

**Conclusion:**

FCH PET/CT appears to be a useful tool for detecting BTs, and further prospective studies are needed to confirm this.

## Introduction

Brown tumors (BTs), or osteitis fibrosa cystica, are rare skeletal manifestations of uncontrolled hyperparathyroidism (HPT). They result from PTH-driven osteoclastic activation and manifest as osteolytic, expansile lesions that can mimic primary bone tumors or metastases (1, 2). Although their incidence has declined (<2% in primary HPT; up to 13% in secondary HPT) (3–5), BTs remain clinically relevant due to diagnostic ambiguity and risk of pathological fractures.

^1^⁸F-fluorocholine (FCH) PET/CT is becoming the leading radionuclide imaging technique for patients with all types of HPT for whom surgery is an option, and will probably be the first-line technique in the near future (6). It also has shown incidental detection of BTs. Its ability to provide whole-body functional and anatomical information positions it as a potentially valuable tool in this context. The novelty of our work lies in emphasizing FCH PET/CT as a dual-purpose imaging modality for both parathyroid and skeletal pathology.

Here, we present three illustrative cases where FCH PET/CT detected BTs, correlating imaging with histology and outcome, and review the available literature.

## Clinical cases

### Case 1

#### Patient history

A 26-year-old woman was referred for evaluation of newly developed pain in her left ankle. Her past medical history was notable only for a neurosurgical intervention in 2009 for a benign brain tumor.

#### Laboratory findings

Biochemical assessment revealed profound abnormalities consistent with severe primary hyperparathyroidism. Serum calcium was elevated to 2.9 mmol/L (reference range: 2.15–2.55 mmol/L), parathyroid hormone (PTH) reached 59 pmol/L (reference range: 1.5–4.2 pmol/L), and 24-hour urinary calcium excretion measured 11.3 mmol/24 h. (reference range: 2.5–7.5 mmol/24 h).

#### Imaging findings

Initial imaging included cervical ultrasonography, which demonstrated a hypoechoic lesion inferior to the lower pole of the left thyroid lobe. Dual-phase ^99m^Tc-sestamibi scintigraphy confirmed a corresponding focus of persistent uptake, suggestive of a parathyroid adenoma. Whole-body bone scintigraphy demonstrated multiple foci of increased uptake involving the occipital bone, jaw, spine, ribs, knees, tibias, and the symptomatic ankle. These findings indicated possible skeletal involvement but remained nonspecific.

Subsequently, preoperative FCH PET/CT was performed to refine localization and guide management. FCH was administered intravenously at a dose of 3 MBq/kg, with no requirement for prior fasting. Imaging consisted of a single static whole-body acquisition performed 20 min post-injection, covering 1–2 bed positions with an acquisition time of 2–3 min per bed. A low-dose CT (100–120 kVp, modulated mAs, 2–3 mm slices) was acquired for attenuation correction and anatomical localization. PET data were reconstructed using an ordered subset expectation maximization algorithm with time-of-flight (OSEM + TOF), on a 256 × 256 matrix with 2–3 mm isotropic voxels and a Gaussian post-filter of 2–4 mm, harmonized to EARL standards. The study was performed on a Siemens Biograph mCT 20 scanner. Lesion uptake was quantified using SUVmax, with values greater than 5 considered to represent high uptake.

**Figure 1:**
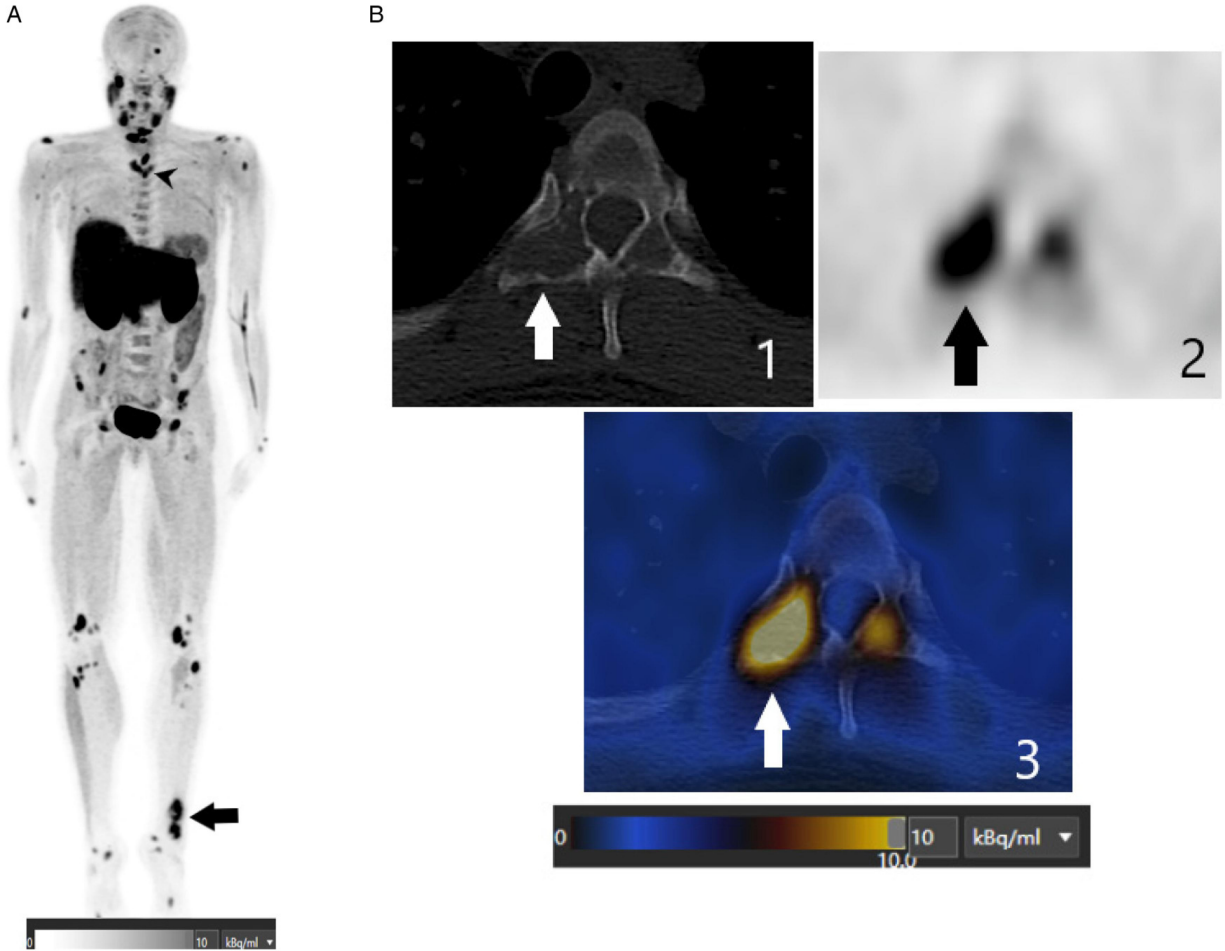
**(A)** This FCH PET/CT maximum intensity projection (MIP) image shows multiple abnormal foci spread throughout the skeleton, including the axial and peripheral skeleton. The black arrowhead indicates a lytic lesion in the Th4 thoracic vertebra. The black arrow indicates a lytic lesion in the left ankle. **(B)** These axial FCH PET/CT images (1: CT image, 2: PET image, and 3: fusion image), centered on the Th4 thoracic vertebra, show intense FCH uptake (SUVmax of 11.5) in the right transverse process, corresponding to a lytic lesion depicted by the arrows.

FCH PET/CT revealed a focal area of intense tracer uptake beneath the inferior pole of the left thyroid gland, measuring 28 mm on CT and displaying a maximum standardized uptake value (SUVmax) of 8.3. This finding correlated with both ultrasonography and sestamibi scintigraphy, confirming a parathyroid adenoma. Beyond parathyroid localization, FCH PET/CT revealed multiple skeletal abnormalities not fully appreciated on prior imaging, including FCH-avid osteolytic lesions in the jaw, vertebrae, ribs, pelvis, long bones, and the left ankle (Figure 1).

#### Histopathology

Surgical excision confirmed a left inferior parathyroid adenoma. Biopsy of the ankle lesion, which had demonstrated intense uptake on FCH PET/CT and osteolytic changes on CT, showed multinucleated giant cells, fibroblastic proliferation, and hemosiderin deposits, consistent with a brown tumor.

#### Treatment and outcome

At five months post-surgery, laboratory parameters had normalized: calcium 2.2 mmol/L (reference range: 2.15–2.55 mmol/L), PTH 1.7 pmol/L (reference range: 1.5–4.2 pmol/L). Follow-up CT demonstrated healing changes in skeletal lesions, including transformation of an initially lytic lesion in the Th4 vertebra into a sclerotic pattern, reflecting bone repair. Clinically, the patient reported resolution of ankle pain and improvement in general well-being.

### Clinical case 2

#### Patient history

A 22-year-old woman was referred for evaluation of progressive visual disturbances. She reported blurred vision and intermittent diplopia over the previous two months. On physical examination, right-sided proptosis was noted, accompanied by mild restriction of upward gaze. Visual acuity remained preserved, and no other neurological deficits were observed.

#### Laboratory findings

Blood tests demonstrated severe primary hyperparathyroidism. Serum calcium was markedly elevated at 3.1 mmol/L (reference range 2.15–2.55 mmol/L), PTH was profoundly increased at 95 pmol/L (reference range: 1.5–4.2 pmol/L), phosphate was reduced at 0.7 mmol/L (reference range: 0.8–1.5 mmol/L), and alkaline phosphatase was elevated at 285 U/L (reference range: 30–130 IU/L), consistent with accelerated bone turnover.

#### Imaging findings

FCH PET/CT was performed as part of the initial diagnostic work-up, using a static whole-body acquisition 20 min after the intravenous administration of 185 MBq FCH on a Siemens Biograph mCT scanner. The scan demonstrated intense focal uptake adjacent to the inferior pole of the left thyroid lobe, consistent with a hyperfunctioning parathyroid adenoma.

**Figure 2:**
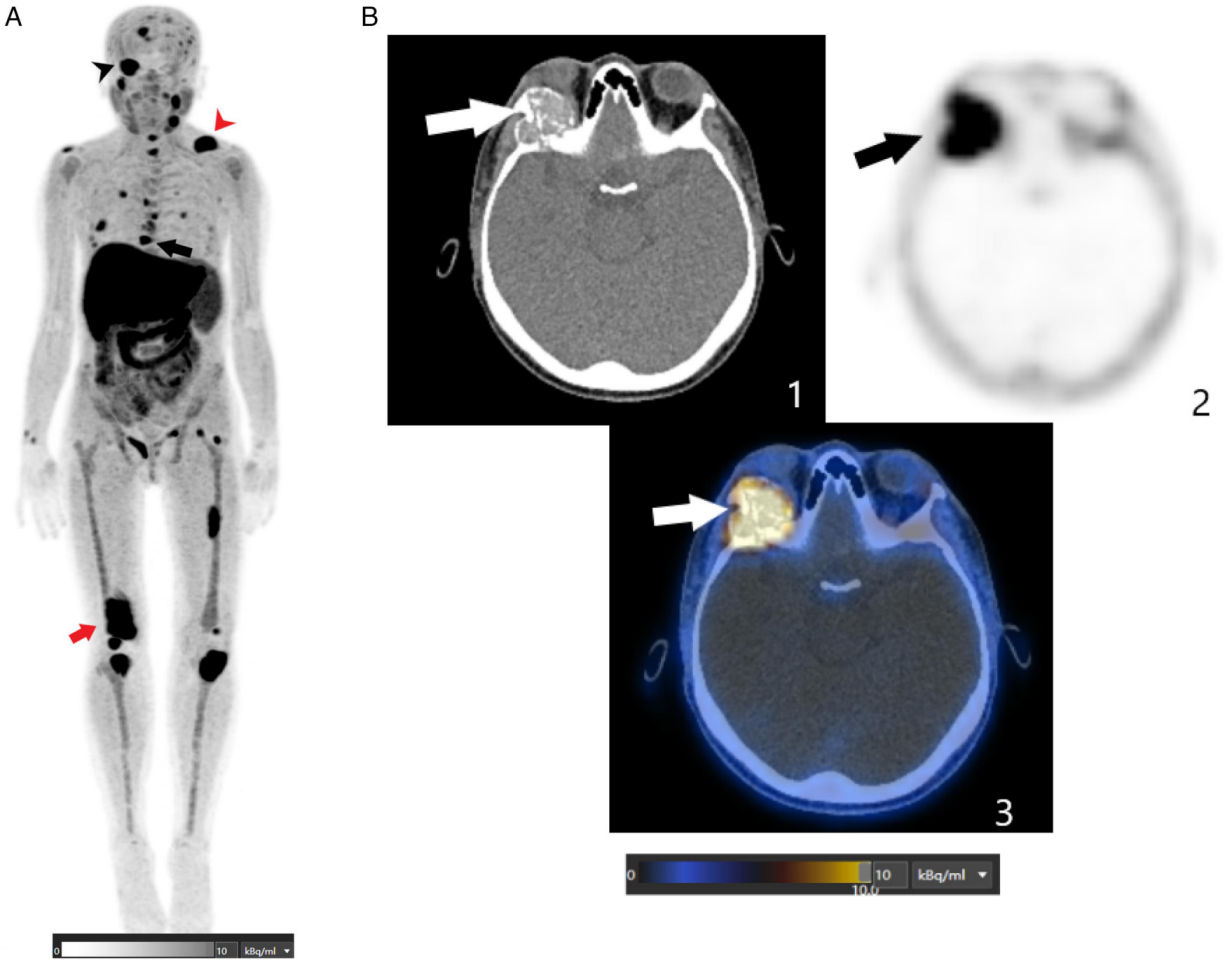
**(A)** This FCH PET/CT MIP image shows multiple abnormal foci spread throughout the skeleton, including the axial and peripheral skeleton. The black arrowhead indicates a hypermetabolic osteolytic lesion involving the right orbital roof and lateral wall, extending into the orbital cavity. The red arrowhead indicates a lytic lesion in the left clavicle. The black arrow indicates a lytic lesion in the T8 thoracic vertebra. The red arrowhead indicates a lytic lesion in the right femur. **(B)** These axial FCH PET/CT images (1: CT image; 2: PET image; and 3: fusion image) are centered on the orbits and show an intense FCH-uptake osteolytic lesion (SUVmax of 13.2) involving the right orbital roof and lateral wall and extending into the orbital cavity (depicted by the arrows).

Beyond parathyroid localization, whole-body imaging revealed multiple FCH-avid skeletal lesions. Maximum intensity projection images showed widespread abnormalities throughout the skeleton. The most striking finding was a large hypermetabolic osteolytic lesion involving the right orbital roof and lateral wall, with extension into the orbital cavity, correlating directly with the patient's ocular symptoms. Additional tracer-avid lesions were observed in the mandible, cervical and thoracic vertebrae, ribs, pelvis, and long bones, including the femurs and tibias (Figure 2).

#### Histopathology

Given the atypical orbital involvement and the need to exclude a malignant process, a biopsy of the orbital lesion was performed. Histopathological examination confirmed the diagnosis of a brown tumor, revealing multinucleated giant cells within a fibroblastic stroma and abundant hemosiderin deposits.

#### Treatment and outcome

The patient was referred for multidisciplinary management. Ophthalmological monitoring was prioritized to address the risk of progressive visual impairment. Calcium-lowering therapy was initiated, and plans were made for definitive parathyroidectomy once metabolic stabilization was achieved.

### Clinical case 3

#### Patient history

A 67-year-old woman presented with several months of progressive back pain and bilateral leg discomfort, accompanied by reduced mobility and difficulty walking. She denied any history of trauma or malignancy. Clinical examination revealed tenderness over the lumbar spine and pelvis, with restricted mobility due to pain.

#### Laboratory findings

Biochemical evaluation demonstrated severe hyperparathyroidism. Serum calcium was markedly elevated at 3.4 mmol/L (reference range 2.15–2.55 mmol/L), PTH was profoundly increased at 172 pg/mL (reference range: 1.5–4.2 pmol/L), and phosphate was decreased at 0.6 mmol/L (reference range: 0.8–1.5 mmol/L). Alkaline phosphatase was significantly elevated at 456 U/L (reference range: 30–130 IU/L), reflecting high bone turnover.

#### Imaging findings

FCH PET/CT was performed using a Siemens Biograph mCT scanner, with a static whole-body acquisition 20 min after intravenous injection of 190 MBq of FCH. The scan demonstrated intense focal uptake inferior to the left thyroid lobe, consistent with a hyperfunctioning parathyroid adenoma.

**Figure 3:**
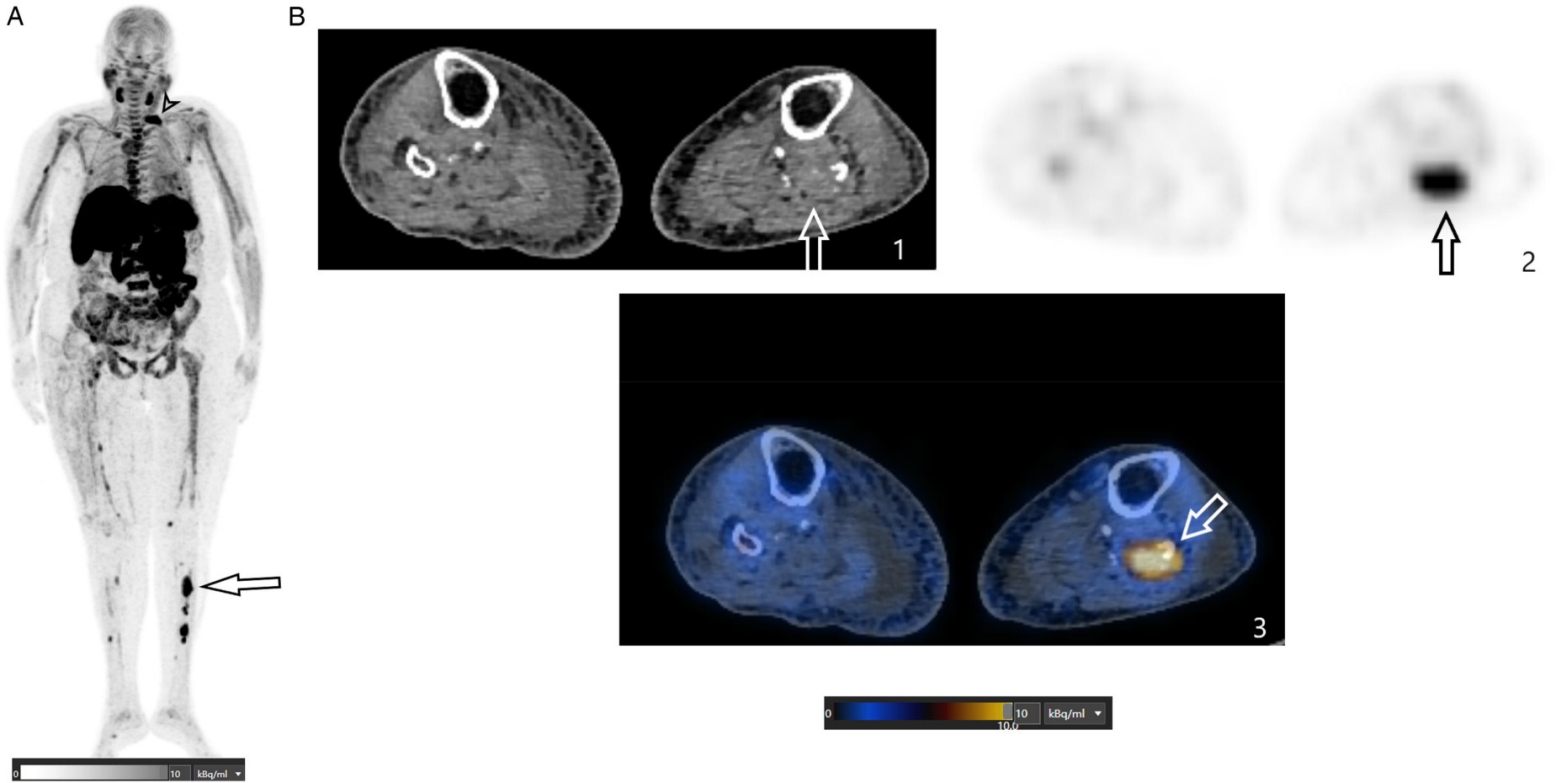
**(A)** This FCH PET/CT MIP image shows multiple abnormal foci spread throughout the skeleton, including the axial and peripheral skeleton. The black arrowhead indicates a lytic lesion in the first rib on the left side. The black arrow indicates a lytic lesion in the left tibia. **(B)** These axial FCH PET/CT images (1: CT image; 2: PET image; 3: fusion image) are centered on the middle third of the leg and show an osteolytic lesion with intense FCH uptake (SUVmax of 9.2) involving the left fibula (indicated by the arrows).

In addition to parathyroid localization, FCH PET/CT revealed numerous hypermetabolic osteolytic lesions across the skeleton, involving the ribs, thoracic vertebrae, pelvis, and long bones (Figure 3).

At the same time, a FDG PET/CT scan was also performed to exclude FDG-avid lesions of a primary tumour and bone metastasis. Similar findings were observed in the bone lesions, but hyperfunctioning parathyroid adenoma was not depicted.

#### Histopathology

Given the extent of skeletal disease and the very high PTH levels, the findings were considered typical for advanced primary hyperparathyroidism complicated by multifocal brown tumors. A biopsy of one tibial lesion was scheduled to exclude malignancy and found results consistent with brown tumors.

#### Treatment and outcome

The patient was referred for urgent endocrinology and surgical evaluation. She received symptomatic treatment including calcium-lowering agents and bisphosphonate infusion to reduce bone resorption. Orthopedic consultation was sought to assess fracture risk and to guide weight-bearing precautions. Definitive treatment with parathyroidectomy was scheduled with the aim of reversing the metabolic abnormalities and stabilizing skeletal disease.

#### Literature review

There were several clinical cases using FCH PET and illustrating its performance. Wang and colleagues in 2014 reported two patients with primary HTP caused by adenomas, each with a BT that showed high uptake on both FDG and FCH PET, and also on MIBI scintigraphy. After surgery, all abnormal uptakes resolved (7). García et al. in 2016 described a patient with secondary HTP and two BTs that showed only moderate uptake on bone scan but intense uptake on 11C-choline PET. Again, after parathyroidectomy, the lesions regressed both clinically and metabolically (8). Zhang-Yin and colleagues in 2019 presented a case of severe primary HTP with about fifteen BTs throughout the skeleton, including the mandible, long bones, and vertebrae. On bone scintigraphy, uptake was heterogeneous, In contrast FCH PET detected all lesions with high intensity, while MIBI remained negative. FCH PET also localized the parathyroid adenoma during the same examination. Postoperatively, lesions demonstrated a rapid decline in uptake, consistent with their benign nature (9).

More recently, Mazurek in 2022 reported a solitary BT in a woman with an adenoma. The lesion showed very high uptake on FCH PET, mimicking a solitary metastasis, but disappeared after adenoma resection (10). The series presented by Jacquet-Francillon and colleagues in 2022 further highlighted the versatility of this technique. In their first case, an adenoma with nine associated BTs was imaged: MIBI localized the adenoma but failed to detect the skeletal lesions, while FCH PET visualized them all, including some non-lytic lesions missed by FDG. In the second case, a parathyroid carcinoma produced ten BTs, all avid on FCH PET, with uptake normalizing after surgical removal. In the third case, another adenoma with seven skeletal BTs was studied; MIBI missed a clavicular lesion that was strongly positive on FCH PET. Postoperative follow-up again showed rapid disappearance of abnormal uptake (11).

## Discussion

FCH PET/CT has emerged as a powerful modality for identifying brown tumors in the context of hyperparathyroidism. In both our cases and published reports, lesions consistently displayed high avidity for FCH, underlining the sensitivity of this method. Uptake was usually intense, with mean SUVmax values frequently exceeding 10, sometimes higher than those of the parathyroid adenoma itself (Table 1). In several series, brown tumors demonstrated SUVmax values ranging from 6 to 15, whereas the culprit adenomas often showed SUVmax values between 4 and 10. Only very small lesions, typically below 10 mm, exhibited lower uptake due to partial volume effects.

**Table 1:** Summary of findings.

Case	Age/sex	BT lesions SUVmax range	BT lesions	BT lesions size range (mm)	BT lesions localizations	Outcome
1	26/F	6.3–13.7	24	5–37	Jaw, spine, femurs, tibias, ankle	Progressive resolution after surgery
2	22/F	7.5–15.9	31	4–41	Orbit, mandible, vertebrae, femurs, tibias	Ophthalmological monitoring Calcium-lowering therapy. Surgery scheduled
3	67/F	5.4–12.5	17	3–29	Vertebrae, pelvis, ribs, tibias	Orthopedic assessment and prevention of fracture. Surgery scheduled

The skeletal involvement detected on FCH PET/CT was frequently multifocal, and in some cases more than 15 lesions were observed. Lesions were located in both the axial and appendicular skeleton, with predilection for the mandible and long bones. Because of this dissemination, whole-body FCH PET/CT is recommended in patients with hyperparathyroidism when brown tumors are suspected, as it allows clinicians to identify lesions at risk of pathological fracture or neurological compromise.

Despite these advantages, several limitations need to be acknowledged. Our series is small, including only three patients, and most of the available evidence remains limited to case reports and small series. Larger prospective studies are necessary to confirm the reproducibility of these findings. Another limitation is the possibility of false-positive interpretations: intense osteolytic lesions can mimic metastases or other giant-cell–rich tumors. In such ambiguous cases, histopathological confirmation remains indispensable. The availability and cost of FCH PET/CT also limit its accessibility compared with more widely available modalities such as bone scintigraphy or sestamibi scintigraphy.

A recurring diagnostic challenge is distinguishing brown tumors from metastatic disease. Both may appear as multiple hypermetabolic skeletal lesions. Certain features, however, favor the diagnosis of brown tumors: association with biochemical evidence of hyperparathyroidism, involvement of atypical sites such as the mandible and clavicles, marginal sclerosis on CT, and slower clinical and radiological progression. Importantly, rapid normalization of uptake following parathyroidectomy is a strong indicator of benignity. Nevertheless, in atypical cases or in the absence of clear biochemical evidence, biopsy remains necessary.

When comparing modalities, FCH PET/CT demonstrates distinct quantitative advantages. FDG PET/CT often shows avidity in brown tumors, particularly in advanced lytic lesions, with SUVmax values ranging from 8 to 20. However, in earlier or less destructive lesions, FCH tends to demonstrate relatively higher uptake than FDG, making it more sensitive in those settings. Sestamibi scintigraphy provides inconsistent results: while adenomas are usually detectable, associated skeletal lesions often remain negative or show only faint uptake. In our review, several cases reported complete absence of MIBI uptake in brown tumors that were clearly visualized on FCH PET/CT. Bone scintigraphy and ^18^F-NaF PET are usually positive due to increased osteoblastic activity, but the uptake pattern is nonspecific and cannot reliably differentiate brown tumors from metastatic disease. In contrast, FCH PET/CT combines detection of both parathyroid adenomas and skeletal lesions with high sensitivity and quantitative detail in a single examination, making it uniquely advantageous in this clinical context.

## Conclusion

FCH PET/CT is a highly sensitive tool for detecting brown tumors in patients with hyperparathyroidism and provides both whole-body skeletal mapping and parathyroid localization. However, its interpretation requires awareness of potential pitfalls, correlation with biochemical findings, and in some cases histopathological confirmation. While our experience and the available literature highlight its promise, larger prospective, comparative and multicenter studies are needed to confirm its diagnostic accuracy, cost-effectiveness, and eventual role in routine clinical practice.

## Data Availability

The raw data supporting the conclusions of this article will be made available by the authors, without undue reservation.
